# Antibiotic Drug screening and Image Characterization Toolbox (A.D.I.C.T.): a robust imaging workflow to monitor antibiotic stress response in bacterial cells *in vivo*

**DOI:** 10.12688/f1000research.51868.1

**Published:** 2021-04-06

**Authors:** Benjamin Mayer, Meike Schwan, Kai M. Thormann, Peter L. Graumann

**Affiliations:** 1 Department of Chemistry, Philipps Universität Marburg, Marburg, Hessen, 35032, Germany; 2 SYNMIKRO, LOEWE Center for Synthetic Microbiology, Marburg, Germany; 3 Institut für Mikrobiologie und Molekularbiologie, Justus-Liebig-Universität Gießen, Gießen, Hessen, 35392, Germany

**Keywords:** Combined workflow, ImageJ2, FIJI, cell shape modelling, R-statistics, machine learning, clustering, image processing, image analysis, automation, drug screening

## Abstract

The search for novel drugs that efficiently eliminate prokaryotic pathogens is one of the most urgent health topics of our time. Robust evaluation methods for monitoring the antibiotic stress response in prokaryotes are therefore necessary for developing respective screening strategies. Besides advantages of common *in vitro* techniques, there is a growing demand for *in vivo* information based on imaging techniques that allow to screen antibiotic candidates in a dynamic manner. Gathering information from imaging data in a reproducible manner, robust data processing and analysis workflows demand advanced (semi-)automation and data management to increase reproducibility. Here we demonstrate a versatile and robust semi-automated image acquisition, processing and analysis workflow to investigate bacterial cell morphology in a quantitative manner. The presented workflow, A.D.I.C.T, covers aspects of experimental setup deployment, data acquisition and handling, image processing (e.g. ROI management, data transformation into binary images, background subtraction, filtering, projections) as well as statistical evaluation of the cellular stress response (e.g. shape measurement distributions, cell shape modeling, probability density evaluation of fluorescence imaging micrographs) towards antibiotic-induced stress, obtained from time-course experiments. The imaging workflow is based on regular brightfield images combined with live-cell imaging data gathered from bacteria, in our case from recombinant *Shewanella* cells, which are processed as binary images. The model organism expresses target proteins relevant for membrane-biogenesis that are functionally fused to respective fluorescent proteins. Data processing and analysis are based on customized scripts using ImageJ2/FIJI, Celltool and R packages that can be easily reproduced and adapted by users. Summing up, our approach aims at supporting life-scientists to establish their own imaging-pipeline in order to exploit their data as versatile as possible and in a reproducible manner.

## Introduction

Bioimage analysis is continuously changing our understanding about the world and how we see our environment. Bacteria are present at µm scale and physiological processes, like cellular signalling events, are even lower than nanometer scale. Cell shape is important for these non-compartmentalized, unicellular organisms. The question of how fast cells grow and divide is connected to tightly regulated intracellular processes like protein-biogenesis from which novel synthesized proteins are translocated along synthesis pathways to their target. Associated proteins play an important role in membrane biogenesis ^1– 3^. Membrane proteins are synthesized by ribosomes and usually co-translationally inserted into the cytoplasmic membrane. In this process, the signal recognition particle (SRP; composed of SRP RNA and of Ffh protein) recognizes the signal sequence of the nascent polypeptide at the ribosome ^4^. This complex is recognized by the SRP receptor FtsY and is delivered to the translocon in the cytoplasmic membrane ^5– 7^. The nascent polypeptide can be inserted into the membrane or translocated across the membrane by the translocon ^8, 9^. Disruption of these processes results in dysregulation of essential networks followed by reduced viability, for instance, via chemically induced stress by antibiotic compounds. Susceptibility towards different antibiotics can vary from organism to organism depending on the mode of action of the compound. To further understand respective mechanisms of cellular stress response in bacteria, morphological feature changes are useful to monitor those *in vivo*. Our goal is to illustrate how an imaging based workflow can be efficiently deployed to monitor these processes as part of an antibiotic drug screening strategy involving cell morphology and viability. Further more, the presented combined workflow shows how to extract valuable information from imaging data in a reproducible manner using classic statistical approaches, as well as unsupervised machine learning algorithms.

## Methods

### Biological model system

Our model organism *Shewanella putrefaciens* CN-32 is a Gram-negative bacteria that occurs in aquatic environments ^10^. Depending on its growth and division cycles, it has approximately 3 µm in length and 1 µm in width at exponential phase. Cell division occurs with peak rates at exponential phase represented by OD _600_ 0.5. In our study, we use markerless insertions at the original gene locus functionally expressing fusion-proteins that are relevant for membrane protein-biogenesis: bacterial signal recognition particle Ffh, its receptor FtsY and ribosomal protein of large subunit L1. In order to monitor druginduced stress responses that affect protein-biogenesis, mVenus is used as a fluorescent protein for fluorescence microscopy. Recombinant strains are cultured at 30°C and 200 rpm in Lysogeny broth (LB) medium without antibiotics. This aspect is beneficial to avoid potential bias induced by compound interaction. To analyze antibiotic stress on membrane biogenesis, the protein synthesis inhibitor puromycin was used (200 *µ*g/ml). Puromycin inhibits translation by preventing the release of the premature nascent polypeptide ^11^.

### Drug screening strategy

Drug screening time-course replicates are taken at different days. Cells are inoculated from an overnight culture and cultured at 30°C and 200 rpm until OD _600_ 0.5. From this batch, 1 ml is sampled into a glass tube as steady-state (NC), 1 ml treated with antibiotics and is continuously incubated with same conditions. A sample of 3 *µl* is taken from the culture at steady state and mounted on slides using 1% ultrapure agarose for reduced background. During imaging of cells at steady-state, cells treated with antibiotics are incubated for a minimum of 30 minutes until image acquisition. Similar to this, samples from treated culture after a minimum of 60 minutes are taken accordingly. Viability assay is additionally performed after timecourse experiments. We assume that cells treated with the compound during the time-course experiment are sublethally impaired in cell-growth and division. In order to do so, cells at steady-state and treated with puromycin are 10 fold serially diluted and distributed on LB-agar plates for further incubation at 30°C overnight and imaged using a Fusion-Gel-Illuminator.

### Image acquisition

**Figure 1.**
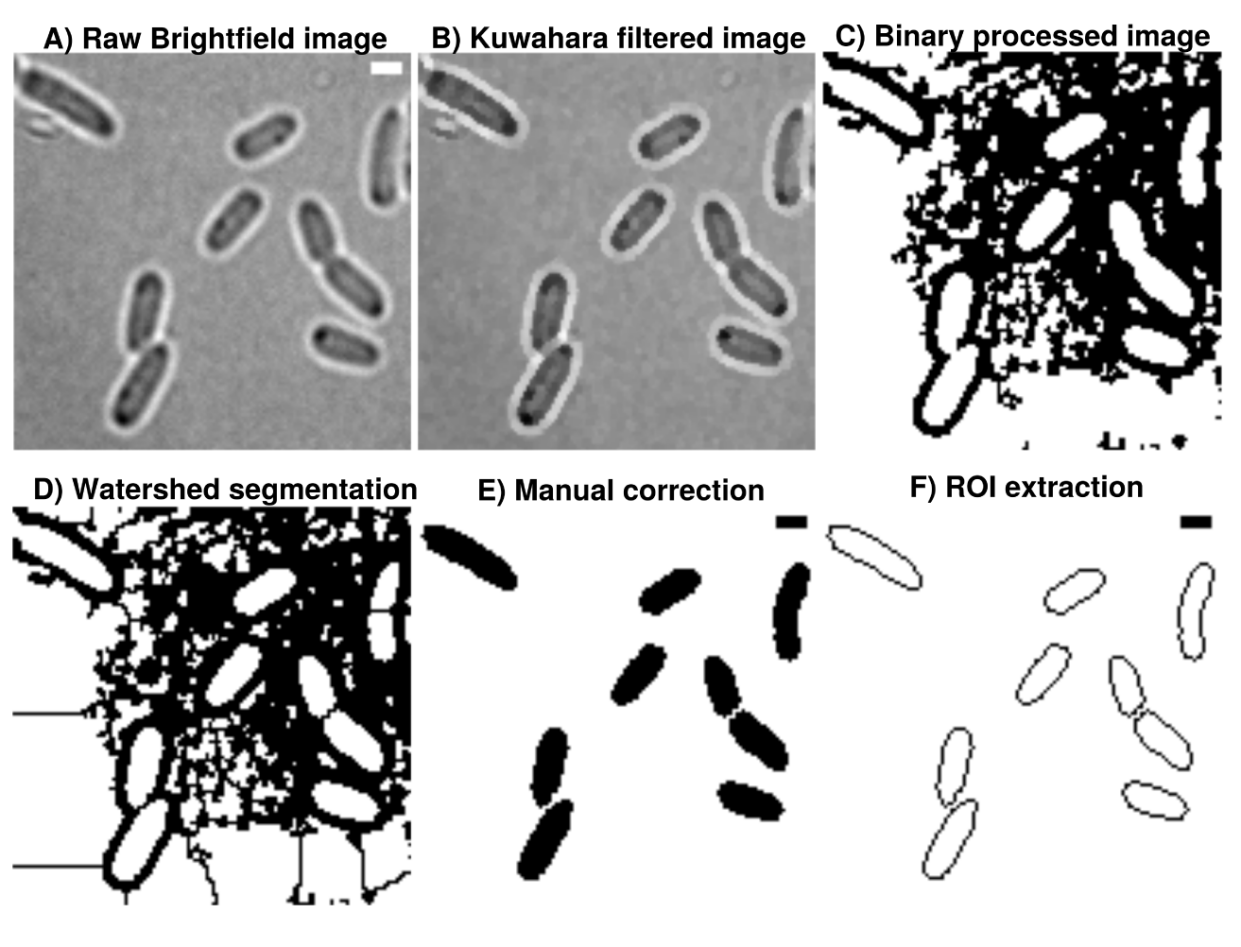
**A**) Brightfield images are transformed from 16-bit into 8-bit and smoothed by **B**) non-linear noise reduction using the Kuwahara filter option in FIJI (sampling window = 2). Noise reduced images are **C**) thresholded using ’Percentile’ thresholding algorithm and **D**) segmented using watershed for cell discrimination. **E**) Resulting binary images are finally corrected manually by correcting potential false positive cells through drawing options. **F**) ROI are extracted from appropriate binary images, stored as individual .zip folders which also involves measurement of cellular areas in an automated manner. This process can be repeated until the the correction is optimal and representative for further analysis. Further analysis always refer to the updated data base and corrections are automatically included when image analysis is reproduced accordingly in Celltool or R-statistics. Scale bar = 1 *µ*m.

**Figure 2.**
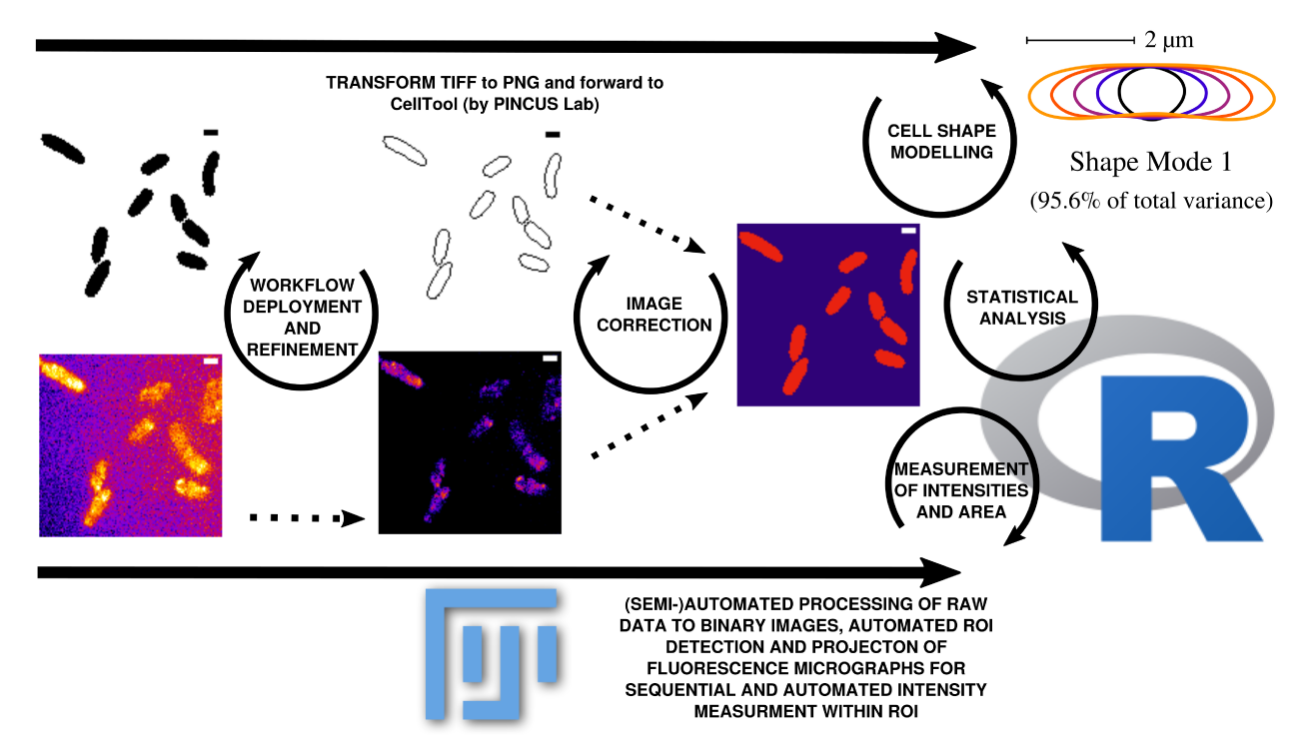
Outline of data processing and analysis steps used in this workflow. Data can be corrected and updated at every stage of the workflow to continuously improve the database until bias is minimized. Scale bar = 1 *µ*m.

Images are acquired using brightfield and photobleaching fluorescence microscopy. In our live-cell imaging pipeline, we use an Olympus IX 71 microscope (100x/NA 1.49/optovar 1.6) customized for slim field microscopy ^12^, with a fast image acquisition conducted by an Andor iXON Ultra EMCCD camera. Brightfield images are acquired using 50 ms exposure time (see Figure 1). Live-cell time-lapse recordings are acquired using 16 ms exposure time. We take advantage of photobleaching steps through continuous slim field illumination until single particles can be localized (see Figure 2). Continuous slim field excitation for photobleaching of the samples is conducted using 514 nm laser line (50%). The microscope setup uses Andor Solis as camera software using at least 2000 frames with integration times of 17.76 ms.

### Image processing and analysis

Image processing in this workflow is conducted using ImageJ2/FIJI ^13– 15^. Binary images are sequentially and automatically annotated, transformed from 16-bit into 8-bit, non-linearily noise reduced using a Kuwahara filter and thresholded using ’Percentile’ thresholding algorithm and segmented using watershed segmentation for cell detection (see Figure 1). Resulting binary images are finally corrected manually using the paint function of ImageJ2/FIJI accordingly (see Figure 1). This process can be repeated until the correction is optimal and representative for further analyses (see Figure 2). Fluorescence time lapse recordings are automatically annotated, cropped after sufficient photobleaching steps and projected using the ’Standard deviation’ method. This method is used for tomographic representations and highlights areas of high fluorescent densities within a region of interest (ROI). Resulting fluorescence micrographs are background corrected using the math function ’substract’. All images are automatically scaled and stored in a database management system that is connected through a set of predefined folder operations within the automation script. Regions of interest (ROI) are automatically detected using ImageJ2/FIJI and further processed using scripts based on macro language implemented in ImageJ2/FIJI (see Figure 1). ROIs are extracted from binary images using the ROI-manager plugin and used as a mask for cell-measures of projected fluorescence micrographs (see Figure 2).

As a result, we receive comma separated value (.csv) tables that are merged using a custom script in R-statistics based on the ’dplyr’ package to organize and merge the tables to a final result-table. Cell shape analysis is conducted using Celltool developed by Pincus lab for cell shape modelling ^16^. Scripts for extraction of polygonal contours, alignment statistical evaluation of probability density of cell areas or curvatures and modelling of shape modes are adapted according to the tutorial from Pincus labs ( https://zplab.wustl.edu/celltool/) (see Figure 2). Statistical evaluation of cell areas collected from ROI and fluorescence measurements mean gray value (mgv) as well as integrated density (IntDen) ( https://imagej.nih.gov/ij/docs/menus/analyze.html) are statistically analyzed with Rstudio using a customized markdown pipeline in R 3.6.1 ^17– 28^ (see Figure 2). To understand the context between mgv and IntDen, is important to know that:

$$\text{Mean Gray Value}\;=\;\frac{\text{Gray values (selection)}}{\text{pixel number}}$$

$$Integrated Density\text{=}Mean Gray Value\times \mathrm{Area}\left(\mu m^{2}\right)$$

Statistical distributions resulting from measurements are tested for normality using the Shapiro-Wilk test. Non-parametric Wilcoxon rank sum test is used to test pairwise for significance (confidence level: 0.95 ; p < 0.05 = * ; p < 0.01 = ** ; p < 0.001 = ***). In order to establish an unsupervised machine learning approach using the ImageJ2/FIJI results tables, Density based clustering of applications with noise (dbscan) R-package is applied to cellular areas and mean gray value with the aim to evaluate clusters that distinguish between cellular amount of fusion proteins (indicated by fluorescence) and cellular areas ^29^.

## Proof of concept

### Cell shape analysis and modelling

**Figure 3.**
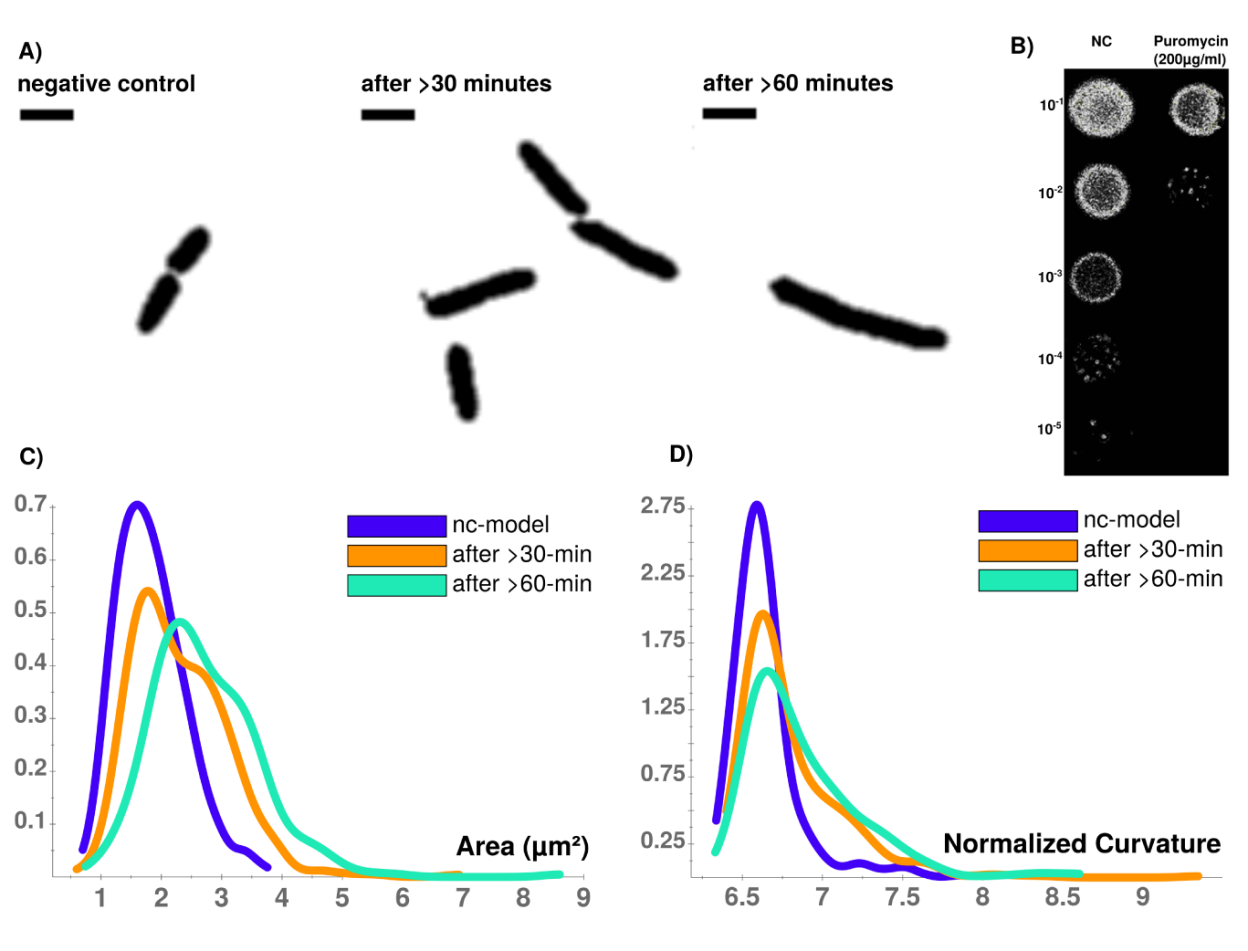
**A**) Time-course of PUR stressed cells at different time points (NC, after >30 min., after >60 min. Viability assay with unstressed (NC) and puromycin stressed cells. **B**) Colonies on LB-agar show drastic effects between serial dilutions of steady-state and puromycin treated cells. Whilst viability is not impaired for steady-state, protein biosynthesis inhibitor puromycin shows sublethal impaired cell growth and division indicated by reduced colony density. **C**) CellTool area comparison shows increasing numbers of larger cells that can be quantified which corresponds to **D**) an increasingly abnormal cell curvature over time after puromycin induced stress. Scale bar = 2 *µ*m.

**Figure 4.**
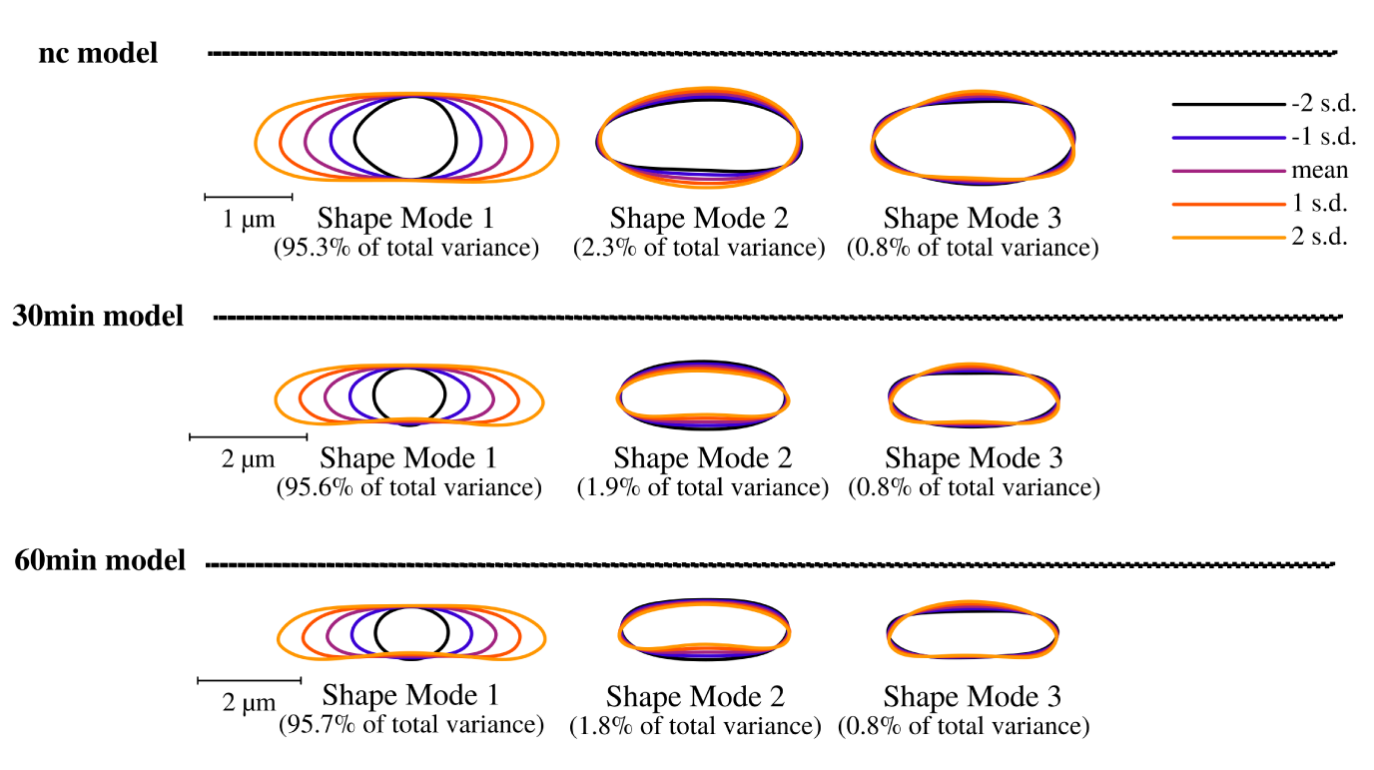
Celltool extraction and modeling of time course imaging results shows generalized model based on its shape variation. Lower limits of explained differences of shape models caused by variation are taken into consideration if not lower than 0.8%. Our results suggest for all time-course acquisitions that mode 1 explains the majority of variation followed by width of the cells represented by mode 2 and 3. Considering the scale bar, the generalized models clearly show that puromycin is indeed affecting the cell length specifically thus cell division might be impaired. These findings correspond to analysis of cellular areas and curvatures.

**Figure 5.**
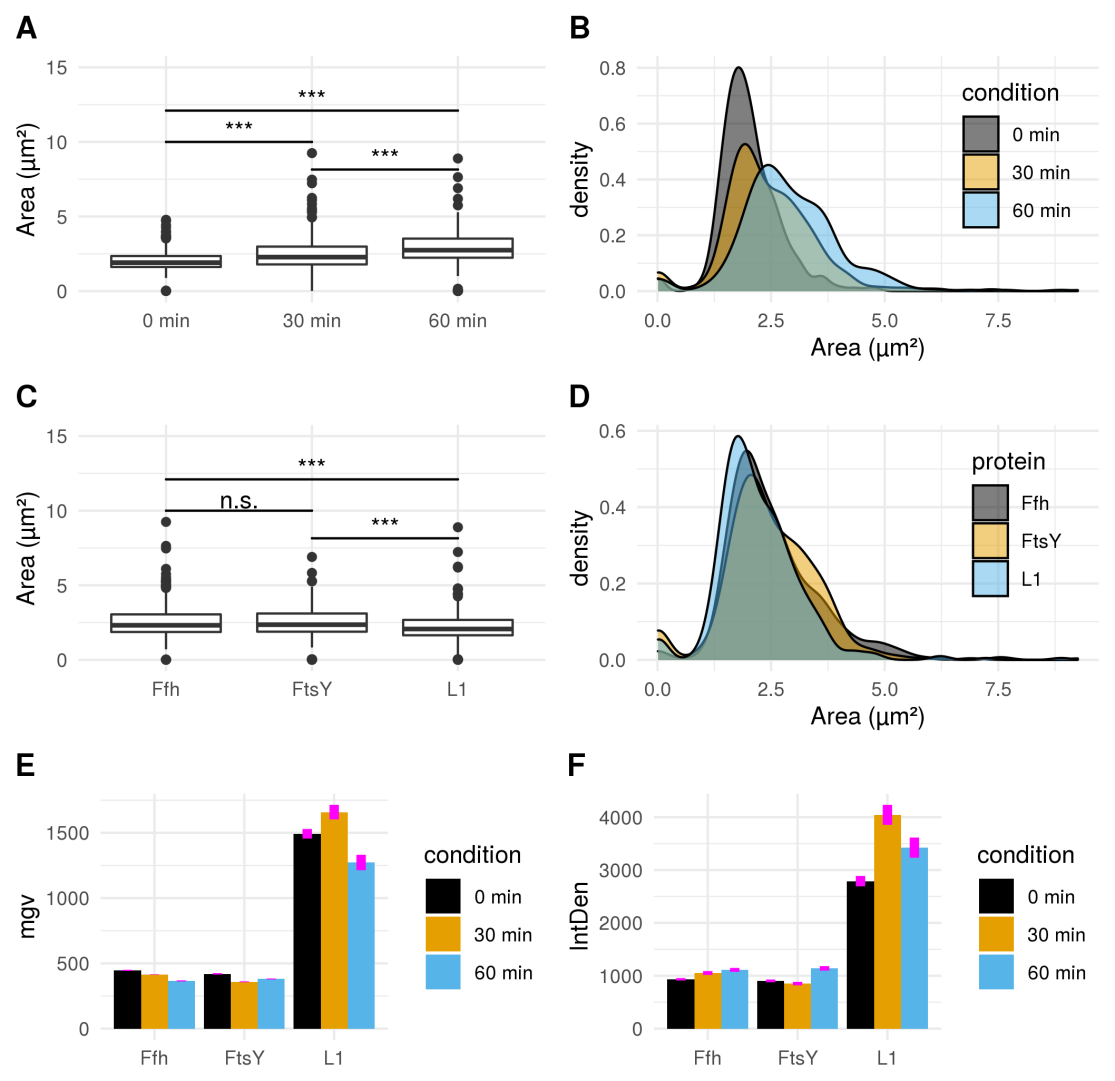
**A**) Boxplot comparison shows increased cell size distribution for all observed fusion proteins over time if stressed with puromycin (0 min: n = 412; 30 min: n = 455; 60 min: n = 338). Extreme value count increases over time for the pooled fusion protein samples. **B**) Probability density of larger cells increases over time for pooled fusion proteins. Results show decreasing number of cells with smaller area indicating that puromycin induced stress results in increase of larger cells for the whole sample. **C**) Cellular areas grouped according to respective strains containing different fusion proteins are differing: Ffh: n = 453; FtsY: n = 371; L1: n = 381. **D**) Probability density plot does not indicate a strong difference if compared between strains of different fusion proteins. **E**) Comparison of mean gray values (mgv) between groups of fusion proteins show slightly decreasing effects over time for Ffh (0 min: n = 131; 30 min: n = 184; 60 min: n = 138) and FtsY (0 min: n = 112; 30 min: n = 160; 60 min: n = 99). L1 (n = 169) appears to increase after 30 min (n = 111) of stress-induction followed by a strong decrease after 60 min (n = 101). Error bars refer to standard error (se). **F**) Integrated density (IntDen) comparison between monitored proteins over time show increasing tendencies over time for Ffh and FtsY. L1 increases after 30 min and decreases after 60 min. Thus IntDen is directly influenced by the area of the cells, it is plausible that IntDen show an overall increase over time compared to mgv, which is not influenced by the cellular area. Error bars refer to standard error (se).

Puromycin treated cells show abnormal cell morphology regarding their size and shape over time (see Figure 3A). Cell shape changes can be modelled with Celltool. Generalized models according to time points show increased variation of cells explained by mode 1 and 2 (see Figure 4), if stressed with puromycin. Corresponding to these findings, probability plots show increasing cell size (see Figure 3C) and abnormal cell morphology reflected by normalized curvature in a quantitative manner during induction with puromycin (see Figure 3D). Depending on the acquisition of the time-interval, cell length increases which indicates cell division stress ^30^. Differences between cellular areas grouped by condition times show clearly a significant time dependent increase (see Figures 5A and B). Although differences of cellular areas appear to be significant as well between L1, Ffh and FtsY if grouped by respective strains, there is no significant difference between Ffh and FtsY. Further more, these findings are strongly influenced by extreme values suggesting that these differences are not a result due to puromycin (see Figures 5C and D). The fluorescence intensity of Ffh and FtsY decreases over time because no new proteins can be translated caused by the protein synthesis inhibitor puromycin and the old proteins are degraded (see Figure 5E and F). From here, it can be only speculated why L1 amount is higher after 30 minutes of induction. One possible explanation could be that the compound does not affect ribosome formation itself. Furthermore, the autocatalytic nature of the ribosomes makes it even more complicated to address ^31^. However, the increased integrated density (see Figure 5F) indicates that changes of the cellular area (in 2D space of course, which is actually a volumetric aspect in 3D) is the decisive parameter to monitor puromycin stress response in our approach. Our assumption that the cellular area is indeed the relevant indicator for puromycin induced stress is further supported by the fact that comparison of cellular areas and curvature with Celltool corresponds to the R-statistics analysis.

### Clustering

**Figure 6.**
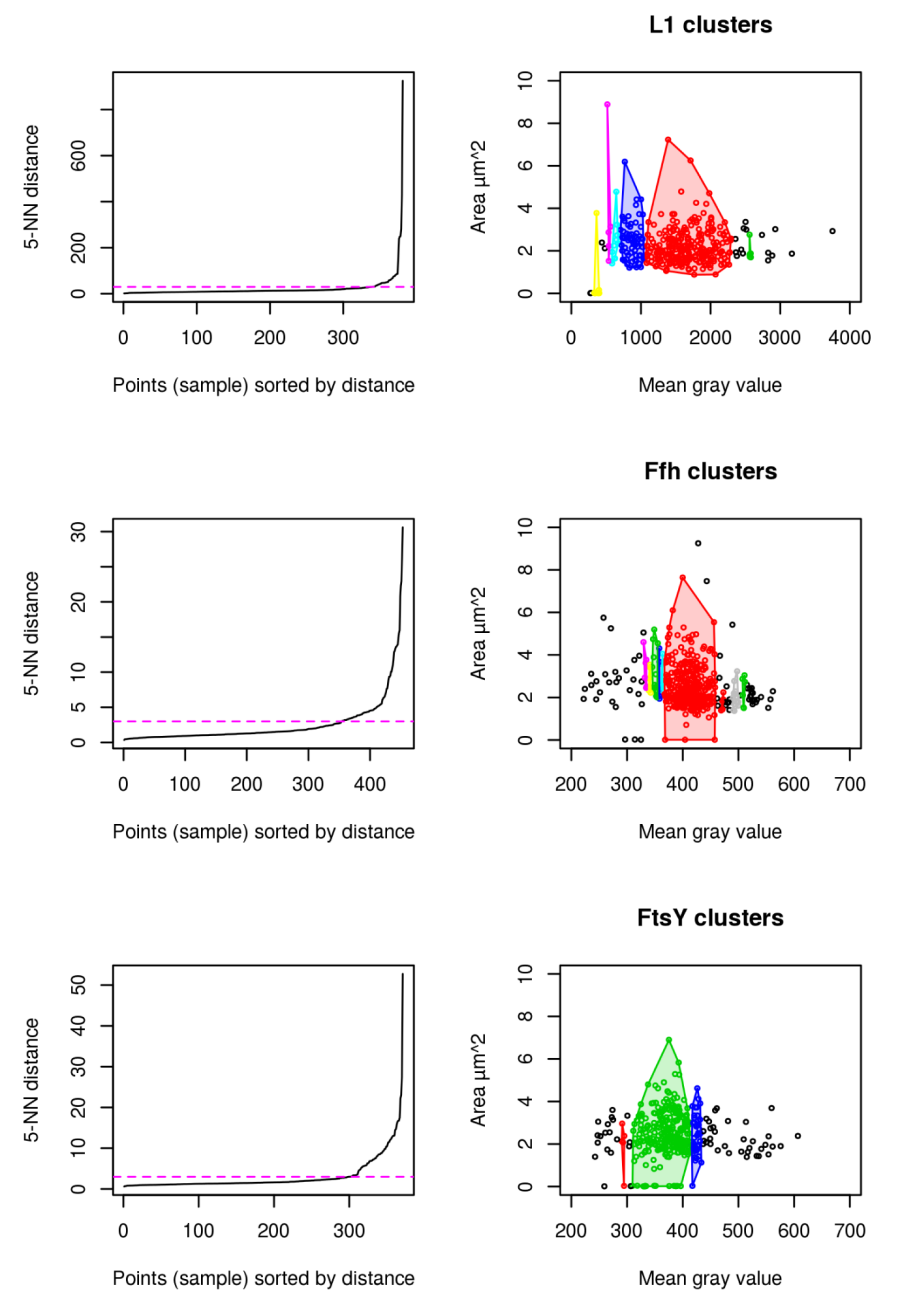
DBSCAN cluster analysis using L1 (n = 381), Ffh (n = 453) and FtsY (n = 371) shows that the cells with largest area do not belong to the cells with the highest amount of protein. 5-Nearest Neighbors (NN) distance plots are used to define the appropriate epsilon values for dbscan and are adjusted manually according to knee of the curve (magenta). Resulting clusters are color coded for discrimination respectively (right).

Results for cluster analysis show that intensity measurements are less powerful to uncover antibiotic stress response in our study compared to abnormal cell grow represented by increased cellular area (see Figure 6). For L1, 6 distinct clusters are identified to which the highest mgv is close beyond 2500 mgv (shown in green). No cell beyond a mgv of 2000 has a larger cellular area than 4 *µ*m ^2^ (see Figure 6 L1). This possibly indicates that higher mgv refers to a non-homogenously distributed population of cells with high L1 amount. It further supports the idea that abnormal cell shape (see Figure 3D and 4) and increased cellular area over time (see Figure 3A) are the relevant indicators for monitoring puromycin induced stress response. In contrast to L1, Ffh shows 10 clusters and FtsY 3 clusters. L1 (red), Ffh (red) and FtsY (green) show one central cluster. However, key finding of the cluster analysis is that cells of respective high protein amount have no enlarged cellular area beyond 4 *µ*m ^2^ for all investigated proteins. It confirms that stressed cells (indicated by enlarged cellular area) do not correspond to increased intensity measurements (compare to Figure 5E and F).

## Conclusions

The A.D.I.C.T. workflow is sensitive enough to monitor time-course drug-screening experiments in a reproducible manner. Binary images are very robust regarding their information content and useful for addressing complex questions involving cell shape modelling at nanoscale levels. However, binary images processed in this study are based on brightfield images and extended manual correction is necessary (see Figure 1). By using more powerful techniques like phase-contrast or specific membrane stains for instance, cell detection would be improved and the effort to manually correct binary images could be decreased further resulting in almost fully automated cell detection. Nevertheless, although we applied very basic image acquisition techniques, it is clearly demonstrated that cell division specific events can be monitored, processed and analyzed using the presented workflow. The advantage of the workflow is that every step can be redeployed and improved starting from the raw data processing to final statistical evaluation using high-level or low-level programming languages. Bias can be reduced by re-deployment and refinement of the database which enhances reproducible dissection and analysis of complex data sets in an automated fashion. The amount of useful information gathered from deploying the A.D.I.C.T. workflow in case of puromycin stress on our model organism is convincing but far away from being fully covered by this article. To sum up, our approach illustrates how powerful very basic imaging techniques can be, if applied with a robust, combined workflow and we hope that it empowers other researchers to take advantage from it for their own research tasks.

## Data availability

Open Science Framework: Test data for A.D.I.C.T. workflow, https://doi.org/10.17605/OSF.IO/FNJ5G ^32^

This project contains the following files:

- Binary images
- Projections
- ROI
- merged results tables (.csv)
- example of brightfield raw images
- example of time-lapse raw recordings

Data are available under the terms of the Creative Commons Attribution 4.0 International license (CC-BY 4.0).

Scripts from this study are available at Github: https://github.com/Image-processing-and-analysis-workflows/A.D.I.C.T.

Archived scripts as at time of publication: http://doi.org/10.5281/zenodo.4616064 ^33^

License: GNU GPL 3.0
