# Supplementary material for: Antibiotic Drug screening and Image Characterization Toolbox (A.D.I.C.T.): a robust imaging workflow to monitor antibiotic stress response in bacterial cells in vivo
Source: F1000Res. 2022 May 17;10:277. Originally published 2021 Apr 6. [Version 3] doi: 10.12688/f1000research.51868.3 (PMC9178280; doi:10.12688/f1000research.51868.3)
Supplement: Peer review history

# Peer review: Reviewer response for version 3

**DOI:** 10.5256/f1000research.133641.r138118

**Published:** 2022-06-08

**Authors:** Janos Kriston-Vizi, Qing Hsuan Ong

**Affiliations:** 1 Bioinformatics Image Core (BIONIC), MRC Laboratory for Molecular Cell Biology, University College London, London, UK; 2 University College London, London, UK

The author addressed our points with the response, thanks.

Is the rationale for developing the new method (or application) clearly explained?

Yes

Is the description of the method technically sound?

Yes

Are the conclusions about the method and its performance adequately supported by the findings presented in the article?

Yes

If any results are presented, are all the source data underlying the results available to ensure full reproducibility?

Partly

Are sufficient details provided to allow replication of the method development and its use by others?

Partly

Reviewer Expertise:

Bioimage informatics, high-content analysis, high-content screening, quantitative cell biology.

We confirm that we have read this submission and believe that we have an appropriate level of expertise to confirm that it is of an acceptable scientific standard.

# Peer review: Reviewer response for version 2

**DOI:** 10.5256/f1000research.77023.r123723

**Published:** 2022-03-02

**Authors:** Janos Kriston-Vizi, Qing Hsuan Ong

**Affiliations:** 1 Bioinformatics Image Core (BIONIC), MRC Laboratory for Molecular Cell Biology, University College London, London, UK; 2 University College London, London, UK

The paper titled as Antibiotic Drug screening and Image Characterization Toolbox (A.D.I.C.T.): a robust imaging workflow to monitor antibiotic stress response in bacterial cells in vivo by Mayer *et al*. reports an image acquisition, processing and analysis workflow to investigate quantitative bacterial cell morphology. The presented workflow includes data acquisition, brightfield image processing with ImageJ/Fiji and statistical data analysis with R.

 A robust automated image processing workflow of high-content brightfield microscopy image dataset is an interesting topic for the community. The authors choice of open-source software ImageJ/Fiji and R is favourable for the academic community as it supports code transparency re-use and reproducibility. The well-commented image processing script is published in the GitHub repository, which adds value to the paper.

 However, the paper only partially delivers the high expectations. The image processing is a 3 steps process: (1) preprocessing using Kuwahara filter, (2) segmentation by calculating the threshold with Percentile algorithm, (3) split virtually merged objects based on concave morphology detection by watershed algorithm.

**Major revisions**

 The limited precision of the Kuwahara-Percentile-Watershed image processing workflow requires the introduction of an additional manual correction step.

- pp5. "Resulting binary images are finally corrected manually using the paint function of ImageJ2/FIJI accordingly".
- Fig 1. "E) Resulting binary images are finally corrected manually by correcting potential false positive cells through drawing options."
- Fig 2. "Although images need to be manually corrected..."
- pp10. "extended manual correction is necessary"

The need for this manual correction of segmentation can be a major limitation of the usability of the workflow for high-content analysis. This limitation is mentioned only marginally. It should be discussed adequately by addressing the following questions:

1. How many images did the screen contain?
2. How many working hours the manual segmentation correction required for the entire image dataset of the screen?
3. Both brightfield and fluorescence images were used in this study. However, it is not clear if brightfield and/or fluorescence images were quantified in figures 3-8. It should be mentioned in figure 3-8 captions which set of raw images (brightfield or fluorescence time-lapse recording) did the data come from.
4. pp5. "Binary images are sequentially" - The image processing pipeline suggests that the sentence must be clarified logically, e.g. replace "Binary images" term with "Raw grayscale images" term.
5. The paper needs more clarification on the image processing and analysis workflow of the fluorescence time lapse images.
6. The role of photobleaching needs to be explained.
7. Only sample raw images are made available. Not all screening image source data underlying the results are available to ensure full reproducibility.

**Minor revisions**

- pp5. "ROIs are extracted from binary images..." - This sentence needs clarification, the "use of ‘Otsu’ thresholding" is unclear.
- pp5. Figure 3. - A dotted arrow seems missing between the top left panel and the right one next to it.

**Typos**

- pp5. "corrected using the math function ’substract’." - Correct ’substract’ to 'subtract'.

Is the rationale for developing the new method (or application) clearly explained?

Yes

Is the description of the method technically sound?

Yes

Are the conclusions about the method and its performance adequately supported by the findings presented in the article?

Yes

If any results are presented, are all the source data underlying the results available to ensure full reproducibility?

Partly

Are sufficient details provided to allow replication of the method development and its use by others?

Partly

Reviewer Expertise:

Bioimage informatics, high-content analysis, cell biology.

We confirm that we have read this submission and believe that we have an appropriate level of expertise to confirm that it is of an acceptable scientific standard, however we have significant reservations, as outlined above.

# Response

**Published:** 2022-04-29

**Authors:** Benjamin Mayer

**Affiliations:** Johann Wolfgang Goethe-University Hospital, Germany

First of all, thank you for the revision of this work. We hope that we can address your points with the following response:

 Although not primarily designed for HCS screening, the usability of the workflow for respective approaches needs clarification. The initial idea was to design a very basic approach that can be easily reproduced by a biologically focused group working on cell-morphology. Therefore, brightfield imaging was used. Fluorescence micrographs can be used to generate more detailed cellular representations, but the general idea was to show how to design a basic imaging pipeline with equipment everyone can use. For this study, moving forward towards creating binary images from fluorescence micrographs is an option but beyond the scope of this work. The primary goal of the workflow is, however, to reduce inconsistencies caused by the experimenter through a streamlined data processing and analysis pipeline that can be fully accessed at every stage of the workflow. To judge whether a cell is divided or not is a source of potential inconsistency harboring the risk of excessive variance, depending on respective impressions by the experimenter. However, the main goal of the workflow is to reduce inconsistencies by maintenance of a documented data basis. Decisions that lead to exclusion of cells that are judged as incorrect (or accidentally not included although correct) can be updated towards re- or disintegration of respective cells within the data basis. The way, how the data basis is processed and analyzed is then updated accordingly. Although HCS was not the primary goal, criteria that are necessary to conduct this would involve different approaches regarding the image quality. In contrast to brightfield imaging, super-resolution imaging offers more possibilities to do so. However, also SR imaging can be affected by technical limitations that might need manual correction. Regarding the presented workflow, also images with better quality that need less correction are possibly better suited for a HCS screen. They could be processed and analyzed with the scripts of this workflow after few modifications.

 ###########################################

 1. How many images did the screen contain?

 ###########################################

 The puromycin data set contains brightfield images for:

 Ffh steady state: 30

 Ffh after 30 minutes of induction: 32

 Ffh after 60 minutes of induction: 43

 FtsY steady state: 33

 FtsY after 30 minutes of induction: 36

 FtsY after 60 minutes of induction: 35

 L1 steady state: 29

 L1 after 30 minutes of induction: 22

 L1 after 60 minutes of induction: 26

 The same number of image sequences that contain the fluorescence images apply accordingly.

 The data set for additional controls using rifampicin (89) and chloramphenicol (79) are only used to confirm Celltool results.

 ###########################################

 2. How many working hours the manual segmentation correction required for the entire image data set of the screen?

 ###########################################

 The manual correction can be conducted approximately within one or two working days for the data set by a trained person. Using drawing tools in IJ allows to quickly eliminate content outside the ROI. However, aim of this workflow is to access these processing steps at every stage if correction of a cell needs attention in order to refine the data set. Thus all downstream processing and analysis of a data basis depends on the maintenance of the data basis, changes are best applied upstream by making decisions (e.g. how many cells?) from the beginning of the processing within the data basis. All further analysis steps can be automated then and are of higher quality, the more accurate the processing of the images is. The purpose of this workflow is not to just push the button and apply something like an autopilot (which is not entirely possible so far, even if the data is super-resolved, artifacts outside the cell in the medium can appear). It is far more an adaptive and at every stage of the workflow fully accessible toolbox that helps scientist who works with imaging based data to minimize bias or inconsistencies of the whole procedure until a representative data set is ready to receive more complex analytical procedures. Analytical features shown in the study give a taste of some procedures that could be implemented based on the extracted information. The scope of this workflow is to focus on extraction, transformation and labeling. Once the corrected data satisfies criteria of acceptance by the investigator, further steps of the protocol can be analyzed quickly within the mentioned working days. However, we are convinced that it is important to get the full control about your data. By looking multiple times over generated data, other interesting observations can be made which could lead (like in the presented study) to further proceedings. In other words, the investment of time in correction pays out in enhanced reproducibility, minimizing bias and structured data transformation for further analysis using Celltool or R. The Celltool modeling was conducted using the most simple approach: binary images based on raw grayscale images. However, using T-projected fluorescence micrographs to extract ROIs is also possible and could be a step forward towards a more fully automated segmentation.

 ###########################################

 3. Both brightfield and fluorescence images were used in this study. However, it is not clear if brightfield and/or fluorescence images were quantified in figures 3-8. It should be mentioned in figure 3-8 captions which set of raw images (brightfield or fluorescence time-lapse recording) did the data come from.

 ###########################################

 Corrected

 ###########################################

 4. pp5. "Binary images are sequentially" - The image processing pipeline suggests that the sentence must be clarified logically, e.g. replace "Binary images" term with "Raw grayscale images" term.

 ###########################################

 Corrected

 ###########################################

 5. The paper needs more clarification on the image processing and analysis workflow of the fluorescence time lapse images

 ###########################################

 (Text also added to caption of figure 3. in the paper)

 Processing of the fluorescence time lapse recordings is conducted on the basis of cropping the relevant frames until single particle level is reached. Analysis of fluorescence micrographs is based on the measurements defined in ImageJ macros and then further processed using an R pipeline with the mentioned packages. The measurements collected using the ImageJ function can be further used for area comparison in combination with mean gray values and integrated densities. ROI that are collected from binary images that are transformed from raw grayscale images are applied to measure the fluorescence micrographs within the respective cell boundaries. This step eliminates signal that is located outside the ROI. The measured value using ImageJ area refers to respective roi and can be compared with the area results from Celltool. Fluorescence micrographs are used to measure intensities that refer to the protein and vitality situation monitored over time. It is important to know that the raw data does not contain useful meta-data. The workflow enables the experimenter to fully control the annotation of the data in a reproducible way. The outcome should be that after applying the workflow to the fluorescence micrographs to receive properly annotated data tables that can be combined for further analysis using R-packages. Intensity measurements directly correspond to protein levels and dynamics. Data can be therefore used to obtain more perspectives about the observed biological process. Cellular stress can be monitored with different variables containing information for fluorescence activity and morphological changes during stress induction. To monitor the same data from different perspectives makes the analysis more robust and allows to explore the cellular stress response in a more detailed and simultaneous manner in an semi-automated manner. This also helps to re-evaluate the analysis in a well documented manner thus the extraction of information and the processing always starts from the raw data (brightfield and fluorescence micrographs). Further modeling for instance becomes easier thus data is well prepared for more complex analysis tasks. Taken together, the workflow allows to evaluate the observed cellular stress response at protein expression levels (fluorescence microscopy) in a simultaneous and interconnected (ROIs based on Bf) manner with stress response observed by cell shape abnormalities (Celltool).

 ###########################################

 6. The role of photobleaching needs to be explained.

 ###########################################

 Photobleaching is used through a constant slimfield illumination that quickly bleaches fusion-protein particles towards a single particle level that can be used for the fluorescence micrographs based on T-projections (T for temporal) described in the paper. Slimfield microscopy is used for single molecule localization microscopy and can be used as a super-resolution technique itself (another article covering this specific aspect is going to be published soon). Photobleaching allows to bleach the fluorescent fusion proteins towards a single particle level. From there, images can be reconstructed, processed and analyzed. However, the scope of the presented workflow aims on the extraction, transformation and labeling aspect. The single particle aspect of the data was already in Mayer et al., 2021. With the presented workflow from this paper, other interesting aspects like the inter-connectivity between cell shape and protein expression based on the data set can be explored. Further more, analysis of the data showed that photobleaching of the particles in the cell can be further exploited to extract useful information about the cell vitality represented by the expression levels of the labeled proteins. Photobleaching varies for the monitored proteins at steady-state and under translation stress represented by respective mean gray values accordingly. 

 ###########################################

 7. Only sample raw images are made available. Not all screening image source data underlying the results are available to ensure full reproducibility.

 ###########################################

 The data set was updated within the repository.

# Peer review: Reviewer response for version 2

**DOI:** 10.5256/f1000research.77023.r93931

**Published:** 2021-09-30

**Authors:** Massimiliano Lucidi

**Affiliations:** 1 Department of Science, Roma Tre University, Rome, Italy

After carefully reading the author's responses, manuscript edits and enrichments, I have no further questions for the authors. Therefore, I consider the proposed work of sufficient scientific quality for indexing in F1000Research.

Is the rationale for developing the new method (or application) clearly explained?

Yes

Is the description of the method technically sound?

Yes

Are the conclusions about the method and its performance adequately supported by the findings presented in the article?

Yes

If any results are presented, are all the source data underlying the results available to ensure full reproducibility?

Yes

Are sufficient details provided to allow replication of the method development and its use by others?

Yes

Reviewer Expertise:

Image processing and bacterial imaging and microscopy

I confirm that I have read this submission and believe that I have an appropriate level of expertise to confirm that it is of an acceptable scientific standard.

# Peer review: Reviewer response for version 1

**DOI:** 10.5256/f1000research.55078.r86409

**Published:** 2021-06-11

**Authors:** Massimiliano Lucidi

**Affiliations:** 1 Department of Science, Roma Tre University, Rome, Italy

The authors of the manuscript entitled “Antibiotic Drug screening and Image Characterization Toolbox (A.D.I.C.T.): a robust imaging workflow to monitor antibiotic stress response in bacterial cells in vivo” presented A.D.I.C.T, a workflow based on brightfield microscopy images that covers aspects of experimental setup deployment, data acquisition, image processing and statistical analysis of the *Shewanella putrefaciens* CN-32 cell morphological alterations upon exposure of puromycin-induced protein synthesis arrest.

 Although the paper is well structured and suitable for the broader readership of F1000Research, in my opinion there are few points that should be revised to increase the quality of this manuscript.

**Major revisions**

1. The authors employed the puromycin antibiotic to analyse bacterial cell morphology modifications. However, this antibiotic is poorly employed to induce morphological alteration in bacteria and has few applications in clinical settings for bacterial infection treatment. On the other hand, many antibiotics induce bacterial cell filamentation at sub-inhibitory concentration such as fluoroquinolones or beta-lactams (Nonejuie *et al*., 2013 ^1^; Htoo *et al*., 2019 ^2^). Why did the authors decide to use puromycin instead of other antibiotics? I suggest applying the CellTool-based method to evaluate bacterial morphological modifications induced by other antibiotics.
2. What is the puromycin minimal inhibitory concentration (MIC) of *Shewanella putrefaciens* CN-32? How much below the MIC is the puromycin concentration used in this work (200 µg/ml)?
3. The most effective thresholding algorithms for bacterial image processing are Otsu or Bernsen (Nichele *et al*., 2020 ^3^). Why did the authors employed ’Percentile’ thresholding algorithm? Please justify this choice in the manuscript.

**Minor revisions**

1. Please substitute “druginduced” with “drug induced” in the Methods section (paragraph: “Biological model system”; line 10).
2. Photobleaching is a phenomenon produced by laser exposure in fluorescence microscopy and it is not a typology of fluorescence microscopy. Please eliminate “photobleaching” in the sentence “Images are acquired using brightfield and photobleaching fluorescence microscopy” from the Methods section (paragraph: “Image acquisition”; line 1) or justify the use of this word in the manuscript.
3. Please add the graph axis titles in Figures 3C and 3D.
4. Please substitute “Further more” with “Furthermore” in the Results section (paragraph: “Proof of concept; Cell shape analysis and modelling”; line 17).

Is the rationale for developing the new method (or application) clearly explained?

Yes

Is the description of the method technically sound?

Yes

Are the conclusions about the method and its performance adequately supported by the findings presented in the article?

Yes

If any results are presented, are all the source data underlying the results available to ensure full reproducibility?

Yes

Are sufficient details provided to allow replication of the method development and its use by others?

Yes

Reviewer Expertise:

Image processing and bacterial imaging and microscopy

I confirm that I have read this submission and believe that I have an appropriate level of expertise to confirm that it is of an acceptable scientific standard, however I have significant reservations, as outlined above.

## References

[1] : Bacterial cytological profiling rapidly identifies the cellular pathways targeted by antibacterial molecules. *Proc Natl Acad Sci U S A* .2013;110(40) : 10.1073/pnas.1311066110 16169-74 10.1073/pnas.1311066110 24046367PMC3791758

[2] : Bacterial Cytological Profiling as a Tool To Study Mechanisms of Action of Antibiotics That Are Active against Acinetobacter baumannii. *Antimicrob Agents Chemother* .63(4) : 10.1128/AAC.02310-18 10.1128/AAC.02310-18 30745382PMC6437480

[3] : Quantitative evaluation of ImageJ thresholding algorithms for microbial cell counting. *OSA Continuum* .2020;3(6) : 10.1364/OSAC.393971 10.1364/OSAC.393971

# Response

**Published:** 2021-08-25

**Authors:** Benjamin Mayer

**Affiliations:** Johann Wolfgang Goethe-University Hospital, Germany

Dear Massimiliano Lucidi,

 Thank you for reviewing our work. We would like to answer questions and highlight improvements we made with this rebuttal letter.

**Major revisions**

**1. The authors employed the puromycin antibiotic to analyse bacterial cell morphology modifications. However, this antibiotic is poorly employed to induce morphological** **alteration in bacteria and has few applications in clinical settings for bacterial infection** **treatment. On the other hand, many antibiotics induce bacterial cell filamentation at sub-** **inhibitory concentration such as fluoroquinolones or beta-lactams (Nonejuie *et al*., 2013;** **Htoo *et al*., 2019). Why did the authors decide to use puromycin instead of other** **antibiotics? I suggest applying the CellTool-based method to evaluate bacterial** **morphological modifications induced by other antibiotics.**

**Answer:** Raw data used in the workflow was generated during a single molecule tracking study about the SRP-pathway (Mayer *et al.* 2021). There, our initial aim was to investigate SRP-ribosome dynamics but during the study, the appearance of abnormally altered cell morphology was observed and subsequently quantified using the A.D.I.C.T. workflow in order to systematically explore these changes. We applied puromycin, rifampicin and chloramphenicol (see figure 6 in the updated version) for inhibitory experiments studying protein-biosynthesis dynamics at single molecule levels. Puromycin is relatively affordable, available and not relevant for medical usage. Data based on puromycin stressed cells serves in this study as a control used for protein-biosynthesis inhibition because it is known to lead to premature peptide chain termination at the peptidyl-transferase site in the 50S ribosomal subunit (reviewed by Aviner in 2020).

**2. What is the puromycin minimal inhibitory concentration (MIC) of Shewanella putrefaciens CN-32? How much below the MIC is the puromycin concentration used in this work (200 μg/ml)?**

**Answer:** With respect to the minimal inhibitory concentration, there is no information available for *S. putrefaciens*. According to general supplier informations, puromycin is weakly active against Gram-negative organisms and for E. coli, 100 µg/ml are recommended. In order to apply sufficient antibiotic stress, we used double of the recommended concentration and applied viability screenings simultaneously to each conducted experiment using serial dilutions to check if the cell response was sensitive enough towards puromycin exposure. Our results show that *S. putrefaciens* cells are susceptible towards puromycin at respective concentration on a sublethal basis (see fig 4B). To our knowledge, results show for the first time, how much puromycin could be used in order to induce sublethal cell-stress in *Shewanella putrefaciens.* However, further improvements of the workflow are also connected to more detailed experiments (e.g. different concentrations) and data-acquisition strategies. We therefore agree that different antibiotics with respective modes of action could be explored in more detail, but that would exceed the scope of this paper showing only a monitoring method focused on delivering useful information about the samples by using very basic and therefore broadly available imaging techniques.

**3. The most effective thresholding algorithms for bacterial image processing are Otsu or** **Bernsen (Nichele *et al., *2020). Why did the authors employed ’Percentile’ thresholding algorithm? Please justify this choice in the manuscript**

**Answer:** Regarding the usage of implemented thresholding algorithms, 'Percentile' was used because it is considered as the most robust tool for this specific purpose. 'Percentile' showed for this particular data set the best background detection without affecting cell areas on average. We added figure 2 to the Area outside cells is corrected manually thus regular brightfield images are used. The decision for this particular thresholding algorithm felt after comparing all thresholding algorithms using the 'Try all' function implemented in FIJI. As mentioned in the text, more advanced microscopy techniques (e.g. phase contrast) could give better images for thresholding and segmentation. Further more and not yet covered by this method paper, projections themselves can be used to detect and to compare drug stressed cell populations according to their shape. We however focused on the most simple imaging technique (brightfield) thus the aim of the study is to show how useful information gain can be established with the presented workflow. We agree with the idea that there are a lot more interesting experiments that could (and should) be conducted to address similar biological questions. We therefore hope that the presented workflow might empowers other investigators in establishing similar pipelines in order to increase reproducibility of their research and exploit their data as versatile and efficiently as possible.

**References:**

*Aviner, Ranen.* *“The* *science of puromycin: From studies of ribosome function to applications in biotechnology.” Computational and structural biotechnology journal vol. 18 1074-1083. 24 Apr. 2020, doi: 10.1016/j.csbj.2020.04.014*

*Mayer B, Schwan M, Oviedo-Bocanegra LM, Bange G, Thormann KM, Graumann PL. Dynamics of Bacterial Signal Recognition Particle at a Single Molecule Level. Front Microbiol. 2021 Apr 30;12:663747. doi: 10.3389/fmicb.2021.663747. PMID: 33995327; PMCID: PMC8120034.*
